# Song's Mast Cell Theory of Acupuncture

**DOI:** 10.1089/acu.2022.0035

**Published:** 2022-10-17

**Authors:** Yong Ming Li

**Affiliations:** ^1^ American TCM Society, New York, USA.; ^2^ Herb Acupuncture Clinic, Bridgewater, NJ, USA.

**Keywords:** Song's theory, mast cell, acupoint, meridian, De Qi

## Abstract

Professor. Jimei Song (1924–1987), from the Liaoning College of Traditional Medicine, first proposed the hypothesis that cutaneous mast cells (MCs) may be responsible for some of the phenomena associated with activation of meridians, acupoints, and De Qi in acupuncture. This was in 1977 and she subsequently published the first investigative report on human subjects. Supported by hundreds of extensive research reports later on, now Song's Mast Cell Theory of Acupuncture is one of the leading theories in acupuncture research. As a scientist and mother, Professor Song belonged to a special generation of female professionals in China. These women were living in a very unique and challenging era. Called “half of the sky” or “bourgeoisie intellectuals,” they faced unbearable difficulties in their lives and their work. The contribution of Professor Song to acupuncture is as significant as the contribution of Ms. Youyou Tu to Chinese herbal medicine. The difference is that Professor Song did not receive any award or significant recognition before she died in 1987. This review provides some background about her life, her contributions, and related publications, as well as a brief review of recent advances on MC mapping and acupuncture based on her MC theory.

## INTRODUCTION

Since the beginning of the last century, many researchers have tried to find the substantive structure of meridians and acupoints. Using anatomical and histologic approaches, these researchers sought to explain the ancient meridian theory and the resulting clinical observations seen in traditional Chinese acupuncture and moxibustion. In the early 1960s, investigations of gross anatomy reported that nerves and blood vessels were more abundant at the acupoints of the human body than at nonacupoints. These studies suggested that nerves and blood vessels play an important role in the structure and function of meridians and acupoints.^1^

**FIG. 1.**
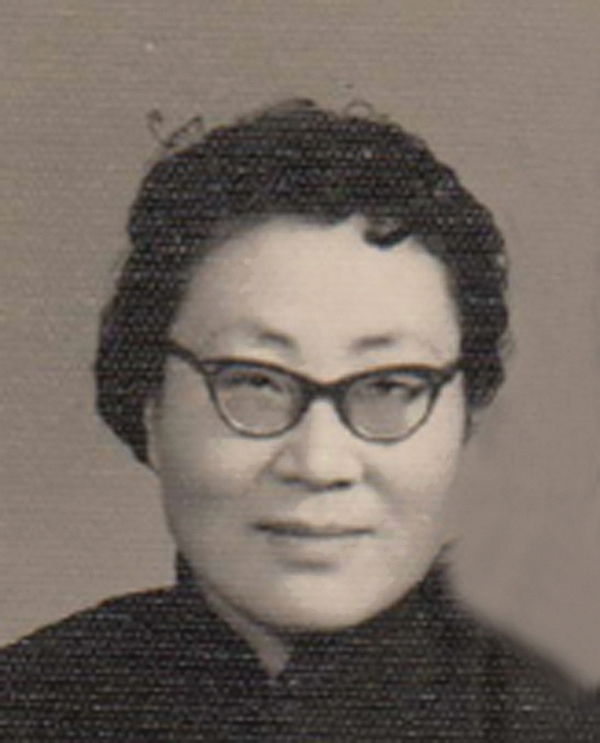
Professor Jimei Song (1924–1987).

A decade later, researchers demonstrated that acupuncture exerted analgesic effects through neurohumoral factors, such as endorphins, resulting in the “humoral theory.”^2^ In addition, during this time, histologic- and cytologic-based studies of acupoints arose as another important area of research in this field. It was this approach that led to the working hypotheses, and ultimately the theory of mast cells (MCs) related to meridians and acupoints. This theory that was first proposed by Chinese Professor Jimei Song (宋继美) in 1977.^3^ See Figure 1.

## BACKGROUND OF SONG'S THEORY

In the late 1970s, acupuncture researchers across China carried out a large-scale study on the sensational [i.e., sensory] phenomenon of meridians. The features of sensational phenomena recorded included patients' feelings after acupoint stimulation, sensation route and speed, blockage by compression, and temperature changes. The route apparently followed the lines of classic meridians and the speed seemed to be different from nerve conduction or blood circulation. The observed skin changes included red or white lines along the meridians, skin papules, patches, petechiaes, or other color changes.^3^ After screening 170,000 people with acupoint stimulation and physical examination, a report was issued in 1977, confirming that “meridian sensations” comprised a generalized phenomenon. The results showed that about one-fifth of the general population had some “sensational phenomena,” and several hundred people were found with multiple “sensitive meridians.”^3,4^ These “meridian phenomena” found in the screening study were similar to the De Qi phenomenon commonly seen in acupuncture treatment. Researchers believed then, as they do now, that a scientific explanation of meridian phenomena could shed the light on the discovery of the substantive basis of meridians and acupoints, which might then have clinical implications.

The results of the meridian study presented a great challenge to biologic and medical scientists in the 1970s, because these phenomena obviously could not be well-explained by the popular neurovascular theory or neurohumoral theories in acupuncture.^5^ The biggest problem at the time was that the speed, sensation, and skin characteristics of meridian sensations were not consistent with the known physiologic phenomena produced by nerves, vascular structures, or circulation systems. The crucial question was: Are there other tissues or cells involved in meridian and acupoint activities?

It was under that particular circumstance that Professor Song proposed the hypothesis that “mast cells are related to meridian and acupoint phenomena,” and her article published in the *Liaoning Journal of Traditional Chinese Medicine* (TCM) in 1977 and 1980 became a milestone in acupuncture research.^3^

## AN INTELLECTUAL, STEPMOTHER, AND UNKNOWN SCIENTIST

Professor Jimei Song (1924–1987) was born into a prominent family from Guangquan, Sichuan, China. Her uncle, Chi-t'ao Tai (Jitao Dai; 戴季陶), was a renowned Kuomintang party member in the Republic of China.^6^ In 1947, Professor Song graduated from Tongji University in Shanghai with a major in biology. Her first job was teaching histology and embryology at the Hunan Xiangya Medical College, which was started as the College of Yale-in-China in 1914. She then transferred to Dalian Medical College, where she met her husband, Zhongming Wang, an anatomist.

At the end of the 1950s, with strong support from the central government and driven by the need for medical education in TCM, a number of TCM colleges were established in China. Professor Song and her husband became the first faculty members as the Liaoning College of TCM (LNCTCM), established in Shenyang in 1958. Professor Song gained valuable experience in medical sciences and acquired substantial knowledge of TCM while working at various institutions. By specializing in anatomy and histology, the couple were a perfect match for teaching and research, and many of their students from the 1960s to the 1980s still remember the excellent lectures they heard from this team today.

Like the Nobel laureate Ms. Youyou Tu, Professor Song belonged to a special generation of female professionals after the establishment of the People's Republic of China in 1949. These female professionals were living in a very unique and challenging era. On the one hand, female scientists and teachers were encouraged to work as hard as men and were regarded as “half of the sky” by Chairman Mao Zedong and the public, while, on the other hand, they had to face a lot of difficulties that male professionals did not face.

During the Cultural Revolution (1966–1976), most highly educated intellectuals were classified as “bourgeoisie” and treated poorly. Except for assigned political tasks, independent research was not encouraged and hardly anything could be done. Professor Song and her husband both graduated from universities with “foreign influences” and specialized in Western sciences, so they fit the mold of “bourgeoisie intellectuals” well.

In addition, Professor Song's family ties to Kuomingtang (later moved to Taiwan) caused her to be labeled as an untrusted intellectual requiring reeducation during many political movements in the 1960s–1970s. Her husband, Mr. Wang, with a similar family background, was not in any better situation. Their normal teaching and research activities were frequently interrupted by these uncontrollable factors.

**FIG. 2.**
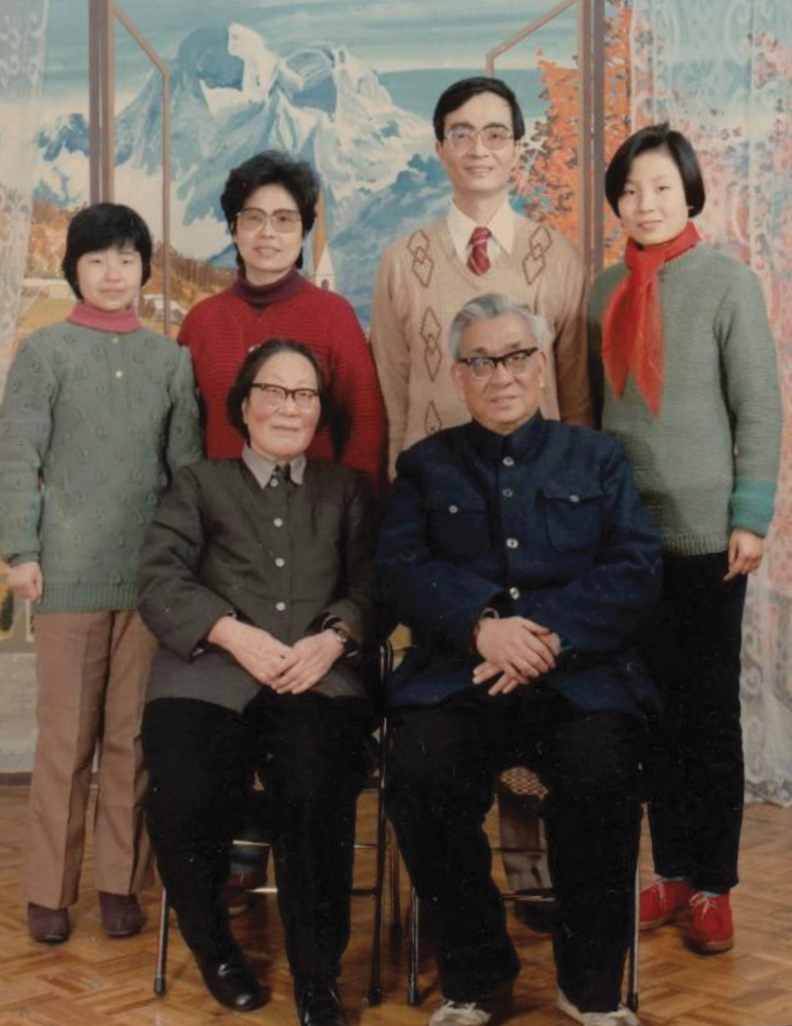
Professor Song and her family. From left to right, front: Professor Jimei Song and her husband, Professor Zhong-Ming Wang; Back: second granddaughter, Wei Wang, daughter-in-law, Hongbing Yao, stepson, Dr. Zi-Mian Wang, and first granddaughter, Ying Wang. This photograph was taken a week before Professor Song died in 1987.

As recalled by Professor Song's stepson, Zi-Mian Wang, PhD, now a retired research scientist from Columbia University in New York City, he thought that Professor Song was “a dove occupying a magpie's nest” (鸠占鹊巢) when he first met his stepmother when he was 8.^7^ Apparently, the stepmother and stepson had some difficult situations over the years, which would not be a surprise for anyone who is familiar with Chinese culture, especially that they were living under a small roof with limited resources. In 1961, the younger Wang ran away from home to Shanghai and traveled more than a thousand miles by train at the age of 15 to start a new life in a boarding school. We can only imagine how much trouble a teenager could cause his parents by doing so. Later, Dr. Wang gradually came to understand the kindness of his stepmother and eventually accepted her fully when his father was taken away from home due to political issues. As an adult, Dr. Wang expressed his increasing professional respect for his stepmother, particularly when he became a scientist himself. Dr. Wang has published several articles and blogs on the motherhood of Professor Song, including an account entitled: “Memories of my Stepmother” in a popular newspaper.^7^ Figure 2 shows Professor Song's family.

Dr. Wang also recalled that his parents were unfairly treated for years, including being sent to the countryside working on farms and being publically criticized in multiple meetings. When school resumed at the end of the Cultural Revolution, the family had to live in the basement of a teaching building on campus due to a limited housing supply.

Even under unbearable pressure, and with very limited resources for research, Professor Song still worked diligently as a female professional and always insisted on a high scientific standard and merit in teaching and research.

At the end of 1963, the official newspaper of Chinese government, *People's Daily*, published a long report on the discovery of the “Bonghan corpuscle” as the anatomical structure of acupuncture meridians by North Korean researcher, Bonghan.^8^ Subsequently, many Chinese scientists tried to repeat the discovery of the Bonghan corpuscle, and it soon became an area of political research for many medical institutes in China.

When Professor Song, was asked by the president of the LNCTCM to jump on the bandwagon, she firmly refused to do any study on that topic, and replied: “I have reviewed over 10 thousand histological slides of human tissue and have never seen anything like a Bonghan corpuscle.” Her refusal led to discomfort for her superior, but, only couple of years later, the president of the institution realized that Professor Song actually saved him from political troubles. As it turned out, the Bonghan corpuscle could not be found in human tissues and has no relevance to meridians, as concluded by many top scientists after 2 years of research.^8^ Bonghan died of suicide after the Korean government issued special postage stamps for his contribution to science. In China, those who supported research on the Bonghan corpuscle were criticized during the Cultural Revolution as having political misjudgment. In addition, Professor Song's brave rejection of that political assignment led to heightened respect from her stepson, as Dr. Wang shared in his memories.^7^

In the meantime, Professor Song and her husband continued their own independent research for many years on meridians and acupoints, seeking evidence to explain acupuncture-related phenomena with anatomical structures and medical sciences. The MC theory was the result of her decade-long efforts.^3^

Professor Song's original work was almost completely forgotten for many years in the acupuncture and research community. The LNCTCM discontinued MC research in 1990s.^6^ Her original publication on the MC hypothesis was rarely cited by researchers and probably never cited in English literature. One of the reasons for Professor Song's relative lack of peer recognition is that her first-of-the-kind investigation on MCs and acupoints in human subjects was not published under her name, but rather under a team name, a common practice at the time.^9^

## PERSONAL NOTES BY THE AUTHOR

Thirty years later, when I started my research on cellular basis of acupoints and meridians, using a dermatopathologic approach in the United States, I soon realized that it was Professor Song, my teacher in medical school, who first proposed the theory of MCs and acupuncture. I went back to the literature and found her first publication on her hypothesis and also the original manuscript of the first investigative report published under a team name, from the storage of the journal's office.^9^ The handwriting confirmed by her stepson proved that Professor Song was indeed the principal investigator and senior author of the first investigation on MCs and acupoints on amputated human limbs.^6^ The result was repeated successfully later by other independent research groups, but no one knew that Professor Song was behind the original publication.

In 2016, I published a review article on MCs and acupuncture and formally proposed Song's Mast Cell Theory of Acupuncture (now called Song's MC Theory for short) in the best journal of acupuncture in Chinese.^10^ In 2018, a symposium on MCs and acupuncture was held in Shanghai to commemorate 40 years of research on MCs and acupuncture. At the Symposium (40^th^ Anniversary of Mast Cell and Acupuncture Research, May 8, 2018) Professor Guanhong Ding, from Fudan University, and I presented reviews of past studies. More recently, Fudan's groups have published 2 detailed reviews in English on the studies of MCs and acupuncture.^11,12^ All of these reviews were built on the first MC hypothesis proposed by Professor Song in 1977.

In my view, the contribution of Professor Song to acupuncture is no less significant than the contribution of Ms. Tu to Chinese herbal medicine. The difference is that Professor Song did not receive any awards or significant recognition before she died in 1987.

## MCs AND MERIDIAN PHENOMENA

In the 1970s, the LNCTCM had no international connection or access to foreign databases for academic literature searches. To access these scientific sources, Professor Song had to take the train for more than 10 hours to find a library at the Chinese Academy of Sciences in Beijing. After extensive research through medical journals, she concluded that “according to the distribution and function of MCs in the human body, it may be responsible for the meridian phenomena.” Accordingly, Professor Song published an article entitled “Mast Cells and Meridian Phenomena,”^3^ in which she cited 16 references, 12 of which were in English, and the most-updated one was published in *Endocrinology* in 1975.^13^

It is amazing that Professor Song could put forward the hypothesis that MCs may be related to meridian phenomena, and that she described a great detail of hypothetical MC responses after acupuncture, solely based on a modern literature search and basic knowledge. It is also interesting to know that, only a few years earlier, Ms. Tu, a Chinese chemist, was searching the ancient literature of Chinese Medicine for an herbal cure of malaria. Ms. Tu found an important clue, which eventually led to the discovery of artemisinin, and won the Nobel Prize.

Professor Song's article is the first publication in the world suggesting that cutaneous MCs participate directly in the phenomena of meridians and acupoints. From then, it has become an important working hypothesis in the study of the acupuncture mechanism. The hypothesis opened up a new field of acupuncture histology and cytology. In the following decades, many laboratories and clinical research institutions have tested this hypothesis through independent investigations. Almost every detail of cellular and biochemical interactions proposed by Professor Song has been confirmed by experimental investigations. Song's MC Theory became the first original theory in acupuncture research named after a Chinese scholar.^10^

Song's MC Theory focuses on MC's role in skin reactions to acupuncture and moxibustion. Given that MCs are major defense cells in the dermis, especially against physical stimuli, it is speculated that MCs release a variety of factors to produce biologic effects. MCs interact with adjacent nerve and vascular tissues and send signals to organs and the central nervous system (CNS). Although the details of Song's MC Theory were limited by the knowledge and technology of the 1970s—the premolecular-biology era—, the basic biologic principles and hypothetical interpretations proposed are still flawless even today.^3,6,10^

**FIG. 3.**
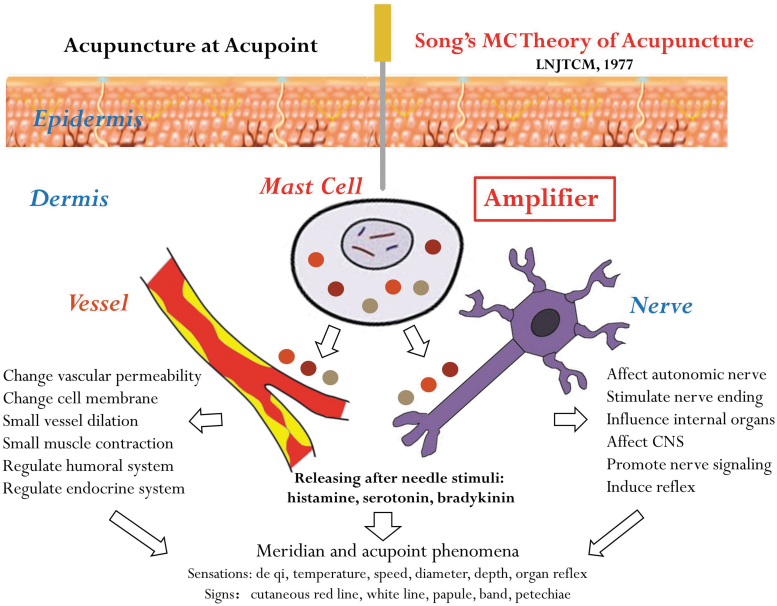
Song's MC Theory of Acupuncture. This illustration is based on the text description in Professor Song's original article published in 1977^3^ and drawn by Yong Ming Li, MD, PhD, LAc, in 2016. (Reprinted with permission.^10^) LNJTCM, *Liaoning Journal of Traditional Chinese Medicine*; CNS, central nervous system.

Professor Song tried to establish a causal relationship between MC's actions and meridian and acupoint phenomena, by providing a reasonable physiologic explanation for De Qi, tingling sensations, skin rashes, and other visible changes that can occur in acupuncture. This is an important addition to understanding the neurohumoral and vascular mechanisms of acupuncture. The core content of Song's MC Theory is illustrated in Figure 3. MCs are common resident cells in the dermis and contain a variety of biologic factors in intracellular granules. After an acupuncture needle penetrates the epidermis, it may stimulate the MCs directly in the dermis to release histamine, serotonin, bradykinin, and proteases, etc., and can lead to a series of effects on the adjacent nerves and blood vessels, which may also induce chain reactions of the CNS, internal organs, and humoral and endocrine systems. More specifically, MCs comprise an “amplifier” in acupuncture responses.^3^ These MC-centered acupuncture responses may explain a large portion of meridian phenomena found in a screening study that cannot be interpreted by neural and vascular mechanisms.

Subsequently, Professor Song organized a multidisciplinary team and carried out a first study of MCs and acupuncture in human. The team published the first research report on MCs in human acupoints in March 1980 entitled, “Preliminary Histological Observations of Mast Cells in Acupoints,” and written by departmental names rather than individual investigators.^9^ It was a small—yet very cleverly designed—experiment. The team located 6 patients who were ready to have amputation surgeries for medical reasons, including 2 arms and 4 legs. With each patient's consent, an experienced acupuncturist identified several acupoints on each limb, and performed acupuncture treatment up to De Qi. After the amputation surgeries, skin tissues from marked acupoints as well as nonacupoints were excised and submitted to histologic study.

Microscopic examinations of the amputated specimens indicated that MCs were present in the dermis of both acupoints and nonacupoints.^9^ The MCs were diffusely distributed in the upper dermis, mainly around small blood vessels, nerve bundles, or nerve endings in groups. As shown in Table 1, the number of MCs in the acupoint areas was significantly higher than in the adjacent nonacupoint areas (mean MCs: 24.40/high-power field at acupoints versus 14.48/high-power field at nonacupoints). The researchers speculated that, when a needle penetrated into the skin, MCs were physically activated and intracellular bioactive substances were released. Accordingly, released histamine, serotonin, and other factors may affect the connective tissue and matrix in the dermis. Nerve endings and other receptors could be activated and send signals to the brain and organs to produce certain meridian sensations. Vasodilation could generate certain signs on the skin surface.

**Table 1.** Comparison of Mast Cells in Acupoints and Nonacupoints

Tissue source	Total MCs	Mean MCs	P-value
Acupoints	366.07	24.40	< 0.001
Nonacupoints	222.56	14.84	

*Note:* Data from Song's publication in 1980. Mean MC represents the number of MCs per high-power field under a microscope. (Reprinted with permission.^9^)

MCs, mast cells.

The finding of different MC densities at acupoints and nonacupoints fully support the hypothesis that MCs play an important role in initiation and amplification of acupuncture stimulation.^9^ In human study, this was of particular significance and pointed a direction for future investigations.^10^ The theory should be considered as a major discovery when searching tissue bases of meridians and acupoints.

## A NEW FIELD OPENED BY SONG'S MC THEORY

Almost immediately after Professor Song's report, many independent research groups started investigations on MCs and acupuncture. Selected articles found in a Chinese database (CNKI.net) follow.

In 1980, Wu et al.^14^ reported the distribution and morphologic characteristics of MCs in the subcutaneous connective tissue of acupoints in rats. Gao et al.^15^ reported the effect of electroacupuncture on the ultrastructure of MCs at acupoints in rats in 1981. In 1985, Zhang^16^ described the ultrastructure of MCs on the human meridian line and their relationship with the neural structure. In 1987, Chen and Zhang^17^ reported an observational study on nerves and MCs in the skin on the presumed meridian lines of mice. In 1989, Lin et al.^18^ published a morphologic and immunohistochemical study of MCs in connective tissue at acupoints in human skin. In 1990, Zhu et al.^19^ reported observing MC counts on the meridian lines in human amputation specimens and MCs in rat meridians, concluding that MC numbers were significantly higher than that in nonmeridian tissue. In 1991, Su et al.^20^ reported that electroacupuncture (EA) on MC degranulation at the *Zusanli* acupoint had effects on pituitary and adrenocorticotropic hormones. In 1992, Zong et al.^21^ studied the effect of EA on fascial MCs in the *Zusanli* area in rabbits.

In the new millennium, *in vivo* studies using animal models continued with some new molecular techniques.

In 2000, Ming et al.^22^ reported that, electrical acupuncture stimulation, more than doubled degranulated MCs in the deep fascia of *Zusanli* in rats, compared to a control group. In 2001, Zhou et al.^23^ noted that EA reduced the inflammatory response caused by trauma, increased the number of MCs, and promoted apoptosis of granulation tissue. In 2003, Li et al.^24^ reported that rats with inflammatory pain treated with EA had increased MCs in acupoint areas and decreased MCs in inflammatory lesions.

Research on MCs and acupuncture continued in the latter part of this decade. In 2007, in a rat arthritis pain model, Lin et al.^25^ found that MC degranulation rates in EA and manual acupuncture groups were significantly higher than in a control group, suggesting that MC degranulation was involved in acupuncture's analgesic effect. Zhang et al.^26^ reported in 2007 that manual acupuncture's analgesic effect in rats was reduced significantly by sodium cromolyn, an MC stabilizer, indicating that MC degranulation was involved in acupuncture's analgesic effect. Luo et al.^27^ reported in 2007 that EA and moxibustion had significantly different effects on numbers, distribution, and degranulation of MCs in rats, and moxibustion had a stronger effect on degranulation than EA. He et al.^28^ reported in 2008 that MC changes in density and granulation rate at auricular and body acupoints correlated with acupuncture effects. Huang et al.^29^ reported in 2009 that nerve blockage at proximal parts of acupoints in rats inhibited acupuncture's analgesic effect significantly but did not affect MC degranulation.

In the next decade, Zhu et al.^30^ reported in 2013 that receptors on the surfaces of MCs can be activated by thermal and mechanical stimulation *in vitro*, which may explain the sensitivity of MCs to acupuncture and moxibustion stimulation. Zeng et al.^31^ reported in 2013 that when they used a collagen model *in vitro*, they found mechanical changes of collagen fibers that may promote activation and degranulation of MCs.

In more-recent years, research on MCs and acupoints continued to be a hot topic, and many research institutes in China have been awarded major research grants to investigate the cellular mechanism of acupuncture. Several leading groups of acupuncture research in China have confirmed or studied MCs' role in acupoints further and have published about this research in international scientific journals and books. These include but not limited to, Ding et al. and Li et al., from Fudan University,^11,12,32^ Wu et al, from the Chinese Institute of Acupuncture and Moxibustion,^33^ Zhao et al, from the Chengdu University of TCM,^34^ and Gong et al., from the Tianjin University of TCM.^35^

Using different animals as experimental models, these independent groups have demonstrated with a broad consensus that: there are abundant MCs at acupoints^36^; acupuncture stimulation can lead to MC degradation and release various biologic factors^37^; specific inhibitors or stabilizers of MCs can reduce the analgesic effect of acupuncture significantly^26,38^; injection of histamine can reproduce some analgesic effects of acupuncture^39^; and animals with genetic defects in MC function show very poor responses to acupuncture.^40^ Finally, an intact nervous system is needed for MCs to mediate acupuncture actions.^29^ These experimental results have supported Song's MC Theory fully.

More-recently, the discoveries of the 2021 Nobel Prize laureates on receptors of temperature and touch have opened the next door for acupuncture research.^41^ Knowing that MCs have TRPV receptors on their surfaces, Zhang et al. and Huang et al. demonstrated with *in vitro* and *in vivo* models that acupuncture needles activate MCs versus TRPV2 receptors.^42,43^ Future research may reveal some thermal receptors on MCs that might mediate the effect of moxibustion.

## CORRELATION OF MC MAPPING AND ACUPUNCTURE SYSTEMS

For a long time, it has been known that MCs are distributed strategically around the body as defense cells for the body's innate immune system.^44^ Mapping of MC distribution in human skin provided strong evidence that MCs indeed could play an important role in acupuncture systems.^45^

**FIG. 4.**
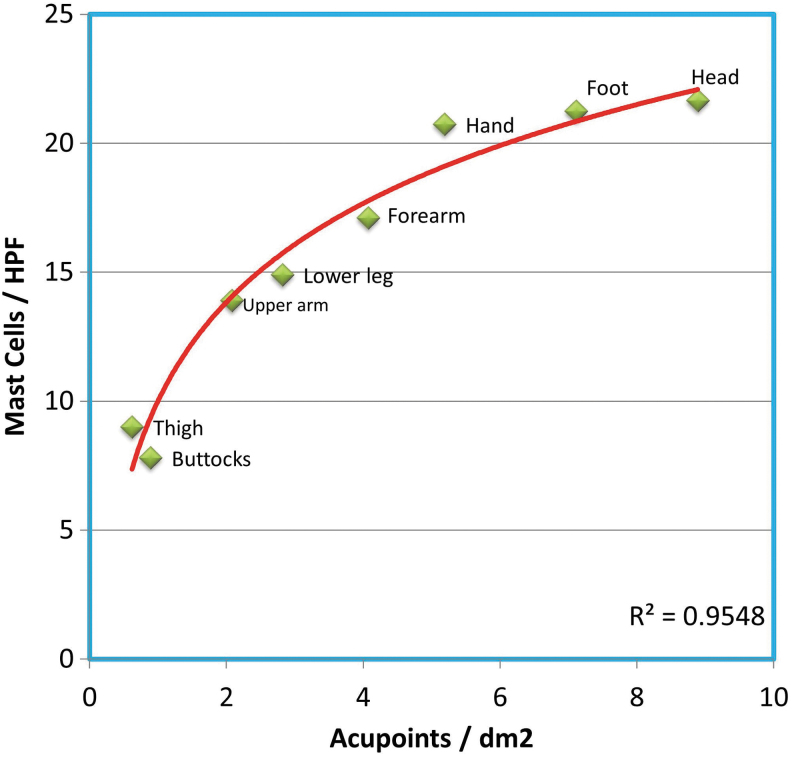
The correlation of dermal MC densities with classic acupoint densities. MCs in human skin biopsies were counted on CD117 immunostained tissue slides under microscopic examination, and data are presented as numbers of MCs per high power field (HPF, 400 × ). The acupoint densities were calculated from 14 classic meridians and are presented as numbers of acupoints per square decimal meter (dm^2^). (Reprinted with permission.^45^)

**FIG. 5.**
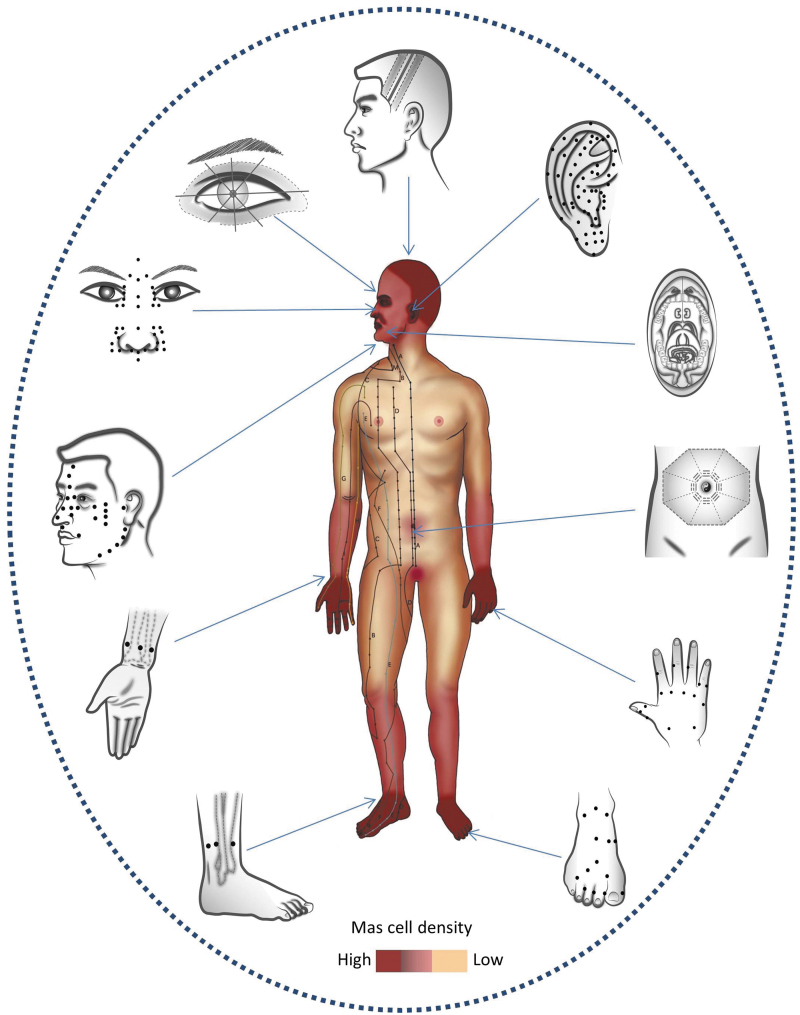
Mast cell (MC) map and acupuncture systems: The distribution of cutaneous MCs shows increased densities approaching peripheral parts and around orifices of the body. All microacupuncture systems are established at MC-enriched special sites [MESS] (with >2 × MCs than the body's trunk), which are shown as darker brown areas. (Reprinted with permission.^46^)

Based on observations of a large number of human-skin biopsies, MC-enriched special sites (MESS) were found at peripheral parts of the body and around the orifices in body surfaces.^45^ Comparative mapping showed that distribution of MC densities is highly correlated with the distribution of the classic acupoints in 14 traditional acupuncture meridians, except for the trunk areas (Fig. 4). Mapping research has also revealed that all microacupuncture systems (newer styles in recent years) were established at MESS areas, including the ears, scalp, peri-eyes, nose, face, wrists, ankles, feet, hands, umbilicus, and mouth (Fig. 5). The conclusion is that densities of cutaneous MCs are highly correlated with acupuncture-suitable areas in either traditional theory or newly developed microacupuncture systems. These findings provided tissue evidence for the neuroimmune basis of acupuncture and suggested that MCs are specific targets for acupuncture stimulation and may serve as tissue markers for acupoints. The findings also fit well with the model of a “bionic principle of acupuncture” that suggests acupuncture needles, like arthropod bites, target MCs in the skin to trigger the defense and healing systems in the body.^46,47^

## CONCLUSIONS

Initially proposed as a hypothesis 45 years ago, Song's MC Theory is now supported by a large body of evidence from basic and clinical research in acupuncture. The theory explains the local cellular responses triggered by acupuncture and many phenomena that could not be explained by neurohumoral and vascular theories previously. MC mapping in the dermis and MESS distribution also provide a reasonable explanation for why superficial needle insertion, including sham acupuncture and various microacupuncture systems, work well for many conditions. There is no doubt that Professor Song was a pioneer and one of the greatest female scientists who made significant contributions to the understanding of acupuncture today.
